# Transcriptomic Response of Huanglongbing-Infected *Citrus sinensis* Following Field Application of a Microbial Fermentation Product

**DOI:** 10.3389/fpls.2021.754391

**Published:** 2021-11-30

**Authors:** Richard D. Lally, Kathleen Donaleshen, Ulalo Chirwa, Katie Eastridge, Wesley Saintilnord, Edward Dickinson, Richard Murphy, Steven Borst, Karina Horgan, Karl Dawson

**Affiliations:** ^1^Alltech Ireland, Dunboyne, Ireland; ^2^Alltech, Nicholasville, KY, United States; ^3^Department of Molecular and Cellular Biochemistry, University of Kentucky, Lexington, KY, United States

**Keywords:** *Citrus*, citrus greening, HLB, huanglongbing, microbial elicitors, fermentation

## Abstract

Huanglongbing (HLB) is considered the most destructive disease in *Citrus* production and threatens the future of the industry. Microbial-derived defense elicitors have gained recognition for their role in plant defense priming. This work assessed a 5% (V/V) microbial fermentation application (MFA) and its role in the elicitation of defense responses in HLB-infected *Citrus sinensis* trees following a foliar application with a pump sprayer. Using a PCR detection method, HLB infection levels were monitored in healthy and infected trees for 20months. Nutrient analysis assessed N, P, K, Ca, Mg, Mn, Zn, Fe, B, and Cu concentrations in the trees. MFA significantly increased Cu concentrations in treated trees and resulted in the stabilization of disease index (DI) in infected trees. Initial real-time qPCR analysis of defense-associated genes showed a significant increase in pathogenesis-related protein 2 (PR2) and phenylalanine ammonia lyase (PAL) gene expression in healthy and HLB-infected trees in response to MFA. Gene expression of PR2 and PAL peaked 6h post-microbial fermentation application during an 8-h sampling period. A transcriptomic assessment using GeneChip microarray of the hour 6 samples revealed differential expression of 565 genes when MFA was applied to healthy trees and 909 genes when applied infected citrus trees when compared to their respective controls. There were 403 uniquely differentially expressed genes in response to MFA following an intersectional analysis of both healthy and infected citrus trees. The transcriptomic analysis revealed that several genes associated with plant development, growth, and defense were upregulated in response to MFA, including multiple PR genes, lignin formation genes, ROS-related genes, hormone synthases, and hormone regulators. This study provides further evidence that MFA may play an important role as a plant elicitor in an integrated pest management strategy in citrus and other agronomically important crops.

## Introduction

Huanglongbing (HLB) is a phloem-limited bacterial disease that infects citrus trees of all kinds, caused by *Candidatus liberibacter* (Jagoueix et al., 1996; Hansen et al., 2008; Duan et al., 2009). It is delivered to the phloem tissue by the Asian citrus psyllid vector and slowly kills trees over a period of several years (Bové, 2006). HLB has devastated the citrus industry in the United States, resulting in severe economic deterioration in affected regions (Dala-Paula et al., 2019; Singerman and Rogers, 2020). Efforts to control and manage HLB have been unsuccessful leading to yearly declines in citrus production. In areas where the disease is not broadly prevalent, management strategies include scouting and destroying infected trees. Aggressive management strategies are recommended for highly infected areas including the use of insecticides, antimicrobials, thermotherapy, antibiotics, and novel therapeutic agents (Davis et al., 2000; Bové, 2006; Das et al., 2014; Gardner et al., 2020). However, effective treatment and a commercially viable cure for the disease have not been found. Defense elicitors in crops have been under investigation as possible solutions to alleviate symptoms of various agronomic diseases. Elicitors upregulate the expression of defense proteins that in turn activate both systemic and local defense responses in treated plants (Montesano et al., 2003). In situations where this activity is observed, the reduction of diseases results in healthier plants and higher yielding crops. In multiple studies, HLB has been observed to interrupt the normal biochemical pathways in citrus. In *Citrus limon*, HLB infection facilitated the downregulation of catalases, chitinases, lectin-related proteins, and miraculin-like proteins (Nwugo et al., 2013). These metabolic changes ultimately lead to increased bacterial spread and susceptibility to other problematic pathogens. Among its repertoire of virulence tools, HLB encodes a salicylic acid hydroxylase, which degrades the plant hormone salicylic acid (Li et al., 2017). HLB also contains peroxiredoxins that increase oxidative stress tolerance, leading to increased HLB fitness *in planta* (Jain et al., 2015, 2018). The routine activation of a plants defense in response to an elicitor could perhaps reduce the success of HLB in citrus.

Microbial fermentation application (MFA), a previously described elicitor program, is formulated with a bacterial fermentation media, yeast cell wall extract, and a Cu component. It has been shown to reduce powdery mildew infection in wheat (Twamley et al., 2019). The authors also concluded that MFA upregulated pathogenesis-related protein 1 (PR 1), pathogenesis-related protein 4 (PR 4), pathogenesis-related protein 5 (PR 5), and pathogenesis-related protein 9 (PR 9) gene expression (Twamley et al., 2019). Upregulation of PR1 is an indicator of increased systemic acquired resistance (SAR) in plants (Ryals et al., 1996) and influences salicylic acid accumulation at the site of infection by various pathogens. The expression of PR1 increases plant tolerance to fungal, oomycete, and bacterial pathogenic challenges (Alexander et al., 1993; Jirage et al., 1999; Zhang et al., 1999). PR5 are a group of thaumatin-like proteins that are also associated with SAR and general defense responses. These are induced in response to various fungal and bacterial challenges (Li et al., 2015b; Chen et al., 2017). Peroxidases, such as PR9, are also widely involved in plant defense (Kawano, 2003) and have been observed in response to bacterial and fungal pathogen infections or exposure.

In this study, MFA was investigated for its potential role in activating citrus defenses against HLB. The previous research in wheat and soybean demonstrates that microbial fermentation products increase the expression of defense networks and aid in the improvement of crop quality parameters or can reduce the burden of disease (Twamley et al., 2019; Schulman et al., 2021; Twamley et al., 2021). If the upregulation of a systemic defense network can be achieved in a similar way in citrus, HLB might be suppressed using the MFA program. Additionally, MFA may affect the plant tissue Cu concentration, as a further mechanism for the restriction of HLB population. To this end, citrus gene expression, nutrient composition, and assessment of routine use of MFA on HLB infection levels quantified by PCR in a field study over the course of 20months were examined. This experiment monitored four treatment groups consisting of an uninfected control, infected trees, uninfected trees with MFA treatment, and infected trees with MFA treatment. Citrus petiole samples were observed routinely throughout the trial to determine the level of HLB infection and how it progressed in response to each treatment. Leaf tissue nutrient analysis was conducted to determine whether nutrient concentrations were influenced in each treatment group. Gene expression of PR2 and phenylalanine ammonia lyase (PAL) was assessed to determine the impact of MFA on defense gene expression between 0 and 8h post-MFA treatment. Samples from the hour 6 time point were used for a transcriptomic assessment using microarray.

## Materials and Methods

### Microbial Fermentation Application

Microbial fermentation application as previously described by Twamley et al. (2019) was a applied as a 5% (V/V) suspension in water for this experiment. MFA contains co-products from bacterial and yeast fermentation processes. Applications were made once monthly to the foliar portion of each tree using a handheld pressurized pump sprayer, for the duration of the experiment. The MFA used in this study was supplied by Alltech Crop Science, KY, United States.

### Field Trial Design

The field trial was conducted for 20months in a privately owned citrus grove in central Florida (Haines City, FL, United States; latitude 28.14297 and longitude −81.69818). A total of 64 *Citrus sinensis* trees were planted in four rows over a 2-acre trial site. To minimize the risk of HLB infection, all the trees were contained in individual plastic protective covers. The trees were split into four groups for the study and were randomly assigned across the four rows. Several trees were removed from the trial due to hurricane damage and unsuccessful infection grafting. The remaining trees were divided across four experimental groups, which included: HLB negative trees (referred to herein as control) *n*=11, HLB positive trees (referred to herein as infected) *n*=17, HLB positive trees with monthly treatments of MFA (referred to herein as MFA+infection) *n*=20, and HLB negative trees with monthly treatments of MFA (referred to herein as MFA) *n*=12. HLB positive groups were infected by grafting HLB positive bud wood onto healthy citrus trees. Grafting was achieved by sourcing infected bud wood from highly symptomatic mature *C. sinensis* trees, which were obtained from a mature symptomatic tree from the same grove. Bud wood of a length of 6–8cm was shaped with a longitudinal cut which was compatible with a cut in the receiving trees. Grafts were firmly secured with 2-cm-wide plastic wrap. HLB infection levels were monitored at 0, 3, 4, 6, 8, and 20months by PCR to determine infection progression and to ensure negative groups remained uninfected. After the grafts were established monthly, applications of a 5% (V/V) MFA treatment were applied to healthy and infected citrus trees. Simultaneously water was applied to a control and the infected control groups. All treatments were applied as a foliar spray and were delivered by pump pressurized equipment, and each treatment was applied until the leaf surfaces were saturated. Each tree received approximately 200ml of each treatment.

### Tissue Sampling and RNA Extraction

After the 15th MFA treatment, samples were taken for qPCR analysis and gene transcriptomic analysis. This gave the trial and disease enough time to establish and receive routine doses of MFA before transcriptomic and nutrient analysis. Seven trees were randomly selected per treatment, and leaf tissue samples were harvested at 0, 2, 4, 6, and 8h post-treatment application. In total, 6 leaves were randomly sampled from each of the selected trees for each time point. Leaves were sealed in plastic zip bags and frozen immediately on dry ice. Tissue samples were transported on dry ice and immediately stored at −80°C at their destination. Leaf tissue was ground to a fine powder under liquid nitrogen, and the frozen powdered tissue was then processed using an RNeasy Plant mini kit (Qiagen, Hilden, Germany) according to the manufacturer’s instructions. An on-column DNA digestion was conducted using the RNase-Free DNase kit (Qiagen, Hilden, Germany) to remove any DNA from the extracted RNA. Purified samples were eluted into a 1.5-ml tube and stored at −80°C until use. Sample quality was evaluated by both NanoDrop (Thermo Fisher Scientific, Waltham, MA, United States) and 2100 Bioanalyzer analysis (Agilent Technologies, Santa Clara, CA, United States) according to the manufacturer’s instructions (Thermo Fisher Scientific, Waltham, MA, United States). Samples with a 230/260 and 260/280 ratio value lower than 2 were rejected and reprocessed. Samples with a RNA Integrity Number (RIN) values >7 were considered acceptable for downstream analysis.

### RT-qPCR Gene Expression Analysis

A total RNA concentration of 2μg was converted to cDNA using the High-Capacity cDNA Reverse Transcription Kit (Thermo Fisher Scientific, Waltham, MA, United States) according to the manufacturer’s instructions. Following the reverse transcription step, the cDNA was diluted 1:20 in nuclease-free water. Target genes encoding for PAL (GenBank accession No. XM_006481430.3) F: 5′-TTGAACTGGGGAGTGA TGGC-3′; R: 5′-CCACTTTGACTTGGGCGTTG-3′ (this study designed with Primer 3) and PR2 (GenBank accession No. XM_015534320.2) FW-F: 5′-ACTTCGCTCAGTACCTTG TTC-3′; R: 5′-GGCAGTGGAAACCTTGATTTG-3′ (Dutt et al., 2015) were considered with 18S (GenBank accession No. XR_003063242.1) F: 5′-GCTTAGGCCAAGGAAGTTTG-3′ R: 5′-TCTATCCCCATCACGATGAA-3′ (Albrecht and Bowman, 2012) as a house keeping gene for examining the relative gene expression of citrus-specific defense indicators.

Reactions were conducted at a volume of 20μl with 10μl Fast SYBR Green Master Mix (Thermo Fisher Scientific, Waltham, MA, United States), 0.1μl F and 0.1μl R primers at a concentration of 10μM each, 8.8μl of nuclease-free water, and 1μl of cDNA template. The quantitative program started with a melt step at 95°C for 20s and then cycled 40 times with an annealing temperature of 60°C for 30s and a melting temperature at 95°C for 3s. Each plate was run with technical duplicates for each sample and a negative control for each target gene. Data were statistically analyzed as 2^−(∆ct)^ data and converted to fold change values for presentation (Schmittgen and Livak, 2008). Fold change values were calculated with the equation 2^−(∆∆ct)^ using the ratio of target gene to control gene. All qPCR analysis was performed on the Applied Biosystems 7500 Fast Real-Time PCR instrument.

### Citrus Transcriptomic Analysis

Leaf samples collected at 6h for the initial qPCR analysis were processed for transcriptomic analysis (*n*=5), using microarray technology. The Affymetrix GeneChip hybridization protocol was used to generate transcriptomic data and was conducted according to the manufacturers protocol for the 3′ IVT PLUS Reagent Kit (Thermo Fisher Scientific, Waltham, MA, United States). Briefly, RNA was isolated as described above and was processed for use with the Affymetrix Citrus genome GeneChip array. Total RNA was prepared to a total reaction concentration of 15μg and used to generate first-strand and second-strand cDNA. Following this, cRNAs were labeled in the presence of biotinylated ribonucleotide analogues (3′ IVT Biotin Label); after purification and fragmentation, a total concentration of 15μg of cRNAs was used in a hybridization mixture containing added hybridization controls. A total of 200μl of the mixture was hybridized on arrays for 16h at 45°C. Post-hybridization, GeneChips were washed and double-stained using the Affymetrix GeneChip fluidics station 450. Chips were then scanned using the Affymetrix GeneChip scanner 2500. Data were exported as CEL files to the Transcriptome Analysis Console (TAC; Thermo Fisher Scientific, Waltham, MA, United States), and data were filtered to include only genes that were expressed to +2 or −2 fold change with a significance threshold of *p*≤0.05 following analysis using the RMA model (Millenaar et al., 2006). All data used for analysis are available in Supplementary File 1.

### Leaf Nutrient Analysis

Citrus leaves, 25–30 per tree, were randomly picked and placed in nitrogen-free tissue sampling bags at the time of the gene transcription analysis. Bags were transported and submitted to Central Florida Soil Laboratory (Bartow, FL, United States). Citrus leaves were washed in 3% hydrochloric acid to remove surface residues and contaminants prior to processing. Samples were prepared using a dry ash method. ICP-OES analysis was used to quantify K, Ca, Mg, Cu, Mn, Zn, Fe, and B (Hansen et al., 2013). N and P concentrations were assessed using a LECO instrument (St. Joseph, MI, United States).

### HLB Progression

Bacterial concentration tests were conducted by submitting leaf tissue samples to Southern Gardens Diagnostic Laboratory (Clewiston, FL, United States). Every tree in the experiment was tested for HLB throughout the study. Once tress were HLB positive as confirmed by a PCR Ct value <33, leaf samples were taken at 0, 3, 4, 6, 8, and 20months into the trial. Month 0 coincided with the first MFA treatment. Six leaves from each tree were sampled and pooled in plastic zip sealed bags and immediately frozen on dry ice. From each sample bag, 100mg of petiole was randomly obtained from across the leaves and placed in extraction buffer. Samples were processed using an automated plant DNA isolation method using Qiagen plant DNA extraction reagents (Qiagen, Hilden, Germany). Assay conditions and primers were prepared according to (Li et al., 2006). Ct values were reported as Ct per 10ngμl^−1^ of petiole DNA. The disease index (DI %) was calculated as previously described by. In short, HLB disease severity was assigned a grade from 0 to 4, where 0=Ct value ≥36.0 (undetectable), 1=32.0≤*C*_t_ value <36 (low HLB infection), 2=28≤*C*_t_ value <32 (moderate HLB infection), 3=24≤*C*_t_ value <28 (high HLB infection), and 4=*C*_t_ value <24 (very high HLB infection). Those values were converted to DI % using the formula described by Yang et al. (2016). Changes in the distribution of disease severity of the infection were measured by examining the changes in the DI in individual trees during the period between 8 and 20months of the trial.

### Statistical Analysis

Statistical testing for HLB concentrations, nutrient, mineral analysis, and qPCR was all computed using R (R-Core-Team, 2020), with all data initially tested with a one-way ANOVA. Where significant differences were detected between groups (*p*<0.05), the data were then subjected to a Tukey’s HSD test, and data achieving a *p*<0.05 were accepted as significantly different. The distribution of the change of disease severity was compared with a Pearson’s Chi-squared test in JMP version 16.0.0 (SAS, Cary, NC, United States). Gene expression data were subjected to independent statistical validation using the TAC console as described in citrus transcriptome analysis section above. Any gene expression results with a value of *p*<0.05 were considered significantly different where fold changes were greater than +2 or less than −2.

## Results

### HLB Progression

**Figure 1:**
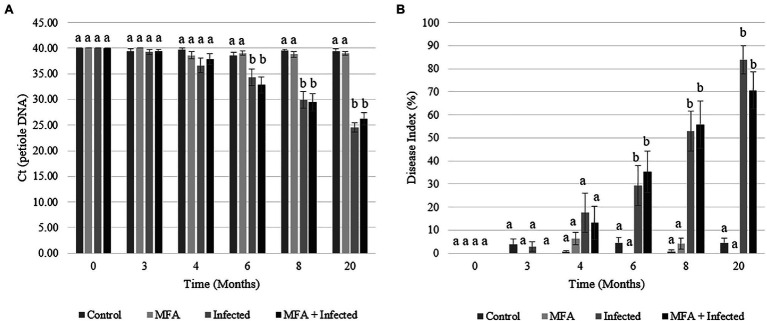
Huanglongbing (HLB) prevalence and disease progression in *Citrus sinensis* over the duration of the field investigation. **(A)** HLB infection as *C*_t_ of petiole DNA measurements was made in infected and uninfected citrus tissue for the duration of the trial. **(B)** Disease index over time. Data represent average disease index rating over time in each experimental group. Letters that are the same within each time point indicate no differences between groups. Error bars represent±SEM.

Huanglongbing prevalence was monitored throughout the experimental period. Uninfected control and MFA trees had no detectable infections for the duration of the trial (Figure 1). The infected and MFA+infected groups showed a steady rise in HLB prevalence over the 6months after graft inoculation (Figure 1A). A numeric difference was observed between both the infected and MFA+infected with the MFA-treated trees scoring a lower average DI rating of 13.3% at 20months although this was not significant (*p*=0.25; Figure 1B).

The ability of the MFA treatment to modulate the progression of the disease and propagation of the infective agent was evaluated during the last 12months of the trial by following the severity of the infections (DI values) in individual trees. Significant changes were recorded in the progression of DI in disease challenged (infected) trees treated with MFA and untreated trees (Table 1). A smaller proportion of trees showed increases in the DI (*p*=0.044), and greater proportion showed decreased DI (*p*=0.008) in the MFA-treated group during this period.

**Table 1:** Disease progression in trees during the period between 8 and 20months of the trial as measured by changes in the severity of infection (DI) in individual trees.

Condition	Treatment	Number of trees showing increased severity of infection (change in DI>0)		Number of trees showing decreased severity of infection (change in DI<0)	
		Fraction	Percent	Fraction	Percent
Unchallenged trees	Control	1/11	9%	0/11	0%
	MFA	0/12	0%	2/12	17%
Infected trees	Infected	12/17^a^	71%	0/17^a^	0%
	MFA+Infected	7/20^b^	35%	5/20^b^	25%
All trees	No MFA Treatment	13/28^a^	46%	0/28^a^	0%
	MFA Treated	7/32^b^	21%	7/32^b^	21%

*Differences in superscripts (a, b) indicate the distribution of the change in disease severity (DI) differs between the MFA treated and untreated groups of trees (p<0.05)*.

### Nutrient Analysis of Leaf Tissue Following MFA Treatment of Control and Infected *Citrus sinensis*

Leaf tissue mineral analysis results indicated that Cu concentrations were significantly higher in trees that received the MFA (Table 2). When compared to the control, both MFA (*p*=0.0063) and MFA+infection (*p*=0.0003) resulted in significantly higher leaf tissue Cu concentrations, and both were also greater when compared to the infected samples (MFA *p*=0.0003 and MFA+infected *p*=0.046). There was a lower Ca concentration in MFA+infected treatment when compared to the control trees (*p*=0.0036). The results also indicated that Mg concentration was lower in the infected group when compared to the control (*p*=0.009) and MFA group (*p*=0.004). The concentrations of the remaining nutrients tested (N, P, K, Mn, Zn, Fe, and B) were unaltered as result of MFA or the HLB disease.

**Table 2:** Leaf tissue nutrient analysis from *Citrus sinensis* after long-term MFA treatment.

	%					Ppm				
Treatment	N	P	K	Ca	Mg	Mn	Zn	Fe	B	Cu
Control	3.22±0.11^a^	0.18±0.01^a^	1.98±0.08^a^	3.76±0.27^a^	0.28±0.01^a^	39.80±6.36^a^	41.80±5.12^a^	88.40±6.23^a^	168.00±16.44^a^	9.8±0.7^a^
Infected	2.98±0.14^a^	0.16±0.01^a^	1.84±0.05^a^	3.22±0.17^ab^	0.22±0.01^b^	38.40±5.10^a^	35.40±0.07^a^	73.80±7.17^a^	185.40±16.26^a^	9.4±0.3^a^
MFA	3.18±0.09^a^	0.20±0.01^a^	1.82±0.07^a^	2.92±0.15^ab^	0.29±0.01^a^	48.00±14.27^a^	44.80±2.42^a^	86.00±9.35^a^	162.80±36.69^a^	32.4±5.4^b^
MFA+Infected	3.04±0.07^a^	0.19±0.01^a^	2.00±0.11^a^	2.44±0.25^b^	0.25±0.01^ab^	37.20±9.90^a^	40.20±4.82^a^	71.00±5.37^a^	184.00±15.37^a^	26.8±6.69^b^

*Data presented represent means of each treatment group for each mineral or nutrient tested. Letters that are the same within each column indicate no differences between groups (n=5). Error is represented as±SEM*.

### Gene Expression Analysis of *Citrus sinensis* in Response to MFA in Control and Infected Trees

**Figure 2:**
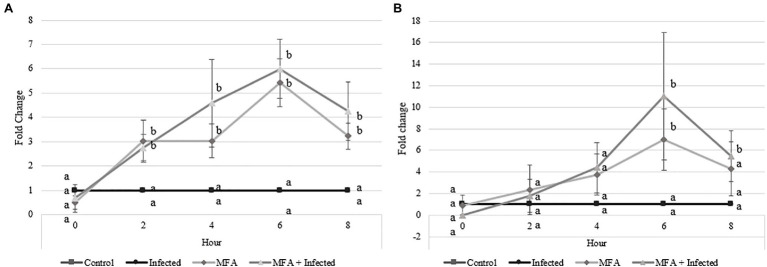
Impact of microbial fermentation application (MFA) on gene expression levels of defense genes in healthy and infected *Citrus sinensis* trees. Data represent gene expression of **(A)** beta-1,3-endoglucanase (PR2) and **(B)** phenylalanine ammonia lyase (PAL) over 5 time points 0, 2, 4, 6, and 8h after MFA treatment (*n*=7). Both treatments were normalized to their respective controls. MFA trees were normalized to control trees, and MFA+infected trees were normalized to the infected group. Letters that are the same within each time point indicate no differences between groups (*p*>0.05). Error bars indicate±SEM.

RT-qPCR analysis of two citrus defense-associated genes was performed in a time point-based assessment (Figure 2). PR2 gene expression increased in MFA and MFA+infected trees 2h after MFA treatment and remained significantly upregulated relatively to the untreated controls at all remaining time points (Figure 2A). The maximum expression level increase for PR2 was observed 6h after the MFA treatment was made, with increases of 5.4-fold (*p*=0.001) and 5.9-fold (*p*=0.001) in MFA and MFA+infected groups, respectively, when compared to the control.

Phenylalanine ammonia lyase expression levels were also significantly increased in MFA and MFA+infected trees (Figure 2B) 6h post-application. MFA and MFA+infection increased to 6.9-fold (*p*=0.01) and 11.0-fold (*p*=0.03), respectively, when compared to untreated controls. PAL expression remained high in the MFA+infected trees 8h after application with a fold change of 5.4 (*p*=0.02).

### Transcriptomic Assessment of Control and Infected Trees in Response to MFA

**Figure 3:**
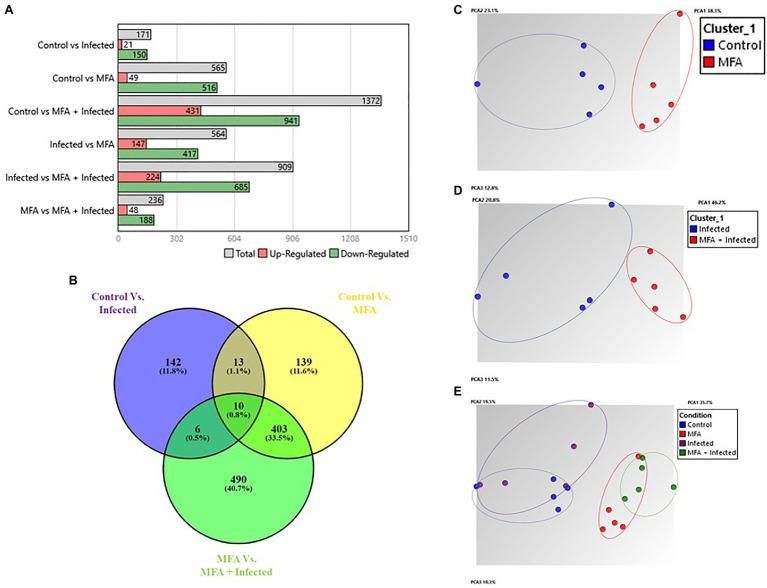
Summary of gene expression data from *Citrus sinensis*. **(A)** Number of significantly differentially expressed genes between different combinations of experimental groups. **(B)** Venn diagram displaying number of intersecting genes between healthy and HLB-infected *Citrus sinensis* following treatment with MFA. Venn diagram was generated by comparing gene expression sets. Venn diagram displays the common tally of genes that are impacted by the experimental conditions. Numbers represent significantly differentially expressed genes (*p*<0.05), and percentages represent the percentage of the total tally. (Oliveros, 2007–2015). Venny 2.1.0. https://bioinfogp.cnb.csic.es/tools/venny/. **(C–E)** Principal component analysis (PCA) plots indicating relationships between differentially expressed genes in MFA-treated samples and control treatments. Oval shapes indicate treatment grouping (control, infected, MFA, MFA+infected). **A**, **C**, **D**, and **E** graphs were generated by the TAC console.

As the highest expression levels for both defense genes following treatment with MFA were observed at 6h (Figure 2), RNA samples from the control, infected, MFA and MFA+infected trees/groups were assessed *via* GeneChip microarray (*n*=5). The results revealed 171 differentially expressed genes between the control and infected group. In both MFA-treated groups, a greater incidence of significantly differentially expressed genes was noted. Five hundred and sixty-five genes were differentially expressed between the control and MFA. Nine hundred and nine genes were differentially expressed between infected and MFA+infected groups (Figure 3A).

These results revealed a pattern of uniquely expressed genes in response to the MFA treatment in both infected and healthy citrus trees. Each group had uniquely expressed genes associated with their experimental conditions. The groups were assessed using the following comparisons: control vs. infected, control vs. MFA, and infected vs. MFA+infected. The control vs. infected group had 142 uniquely expressed genes between the groups representing 11.8% of the gene expression differences. Control vs. MFA had 139 genes that were unique to that group. The infected vs. MFA+infected contained 490 unique genes. There were 403 genes that were differentially expressed and were shared between the two groups MFA vs. MFA+infection and control vs. MFA, indicating a cluster of unique genes regulated by MFA (Figure 3B).

Principal component analysis (PCA) presented in Figure 3 reveals clustering of distinct groupings of the MFA-treated trees, with MFA-treated clustering together and the untreated groups being completely distinct.

### Analysis of Control vs. Infected

The set of genes reported when the control was compared to the infected citrus samples revealed 171 differentially expressed genes, 150 of which were upregulated and 21 were downregulated in infected trees when compared to the control. The range of upregulation was recorded from 2 to 11.43 fold and −2 to −5.03 fold change for the downregulated genes. The top 10 upregulated and downregulated genes are presented in Table 3, and the full comparative set is available in Supplementary File 1. Among the known genes, there was a number that may reveal the impact of HLB on various citrus metabolic processes. Genes associated with early nodulin-like protein 3-like gene, Kinesin-4-like, gibberellin regulated protein, probable polygalacturonase-like, trans-resveratrol di-O-methyltransferase-like, and probable leucine-rich repeat receptor-like protein kinase At1g68400-like were all upregulated. Indicators of defense and stress responses such as peroxidase 30-like, protein Aspartic protease in guard cell 1-like, hypersensitive-induced response protein 2-like, phenylalanine ammonia-lyase-like, and miraculin-like and thaumatin-like protein-like were also upregulated. Genes that may be involved in carbohydrate accumulation such as sucrose synthase 6-like, expansin-A4-like, extensin-2-like, and cellulose synthase-like protein G2-like were also upregulated. Among the downregulated genes were hormone-related genes like auxin-responsive protein IAA29-like, brassinosteroid-regulated protein BRU1-like, and ethylene-responsive transcription factor ERF023-like. Fe(II) transport protein 3, chloroplastic-like, adenylate isopentenyltransferase 3, chloroplastic-like, xyloglucan endotransglucosylase/hydrolase protein 22-like, probable xyloglucan endotransglucosylase/hydrolase protein 23-like, ATC protein, and serine/threonine-protein phosphatase 6 regulatory ankyrin repeat subunit C-like were also impacted. Several genes are currently unknown or were hypothetical proteins including the highest upregulated and downregulated genes (Table 3).

**Table 3:** A sample of the differentially expressed genes in the control vs. infected groups.

Gene ID	Description	Fold change	*p*
*Upregulated*			
Cit.10009.1.S1_s_at	Unknown	11.43	0.0281
Cit.15054.1.S1_at	Hypothetical protein	11.07	0.0067
Cit.7276.1.S1_at	Uncharacterized LOC102630205	10.49	0.0042
Cit.23470.1.S1_s_at	Uncharacterized LOC102625551	9.73	0.0064
Cit.7727.1.S1_at	Early nodulin-like protein 3-like	9.56	0.0020
Cit.20586.1.S1_at	Unknown	8	0.0383
Cit.30043.1.S1_at	Unnamed protein product homologue (*Arabidopsis thaliana*)	7.68	0.0104
Cit.7736.1.S1_at	Unnamed protein homologue (*Arabidopsis thaliana*)	7.68	0.0104
Cit.29883.1.S1_a_at	Kinesin-4-like	6.59	0.0097
Cit.681.1.S1_x_at	Unknown	6.09	0.0203
*Downregulated*			
Cit.13366.1.S1_at	Chalcone synthase homologue (*Cardamine amara*)	−5.03	0.0297
Cit.5973.1.S1_s_at	Serine/threonine-protein phosphatase 6 regulatory ankyrin repeat subunit C-like	−4.67	0.0182
Cit.17562.1.S1_at	Serine/threonine-protein phosphatase 6 regulatory ankyrin repeat subunit C-like	−3.93	0.0205
Cit.31451.1.S1_s_at	ATC protein	−3.34	0.0021
Cit.17724.1.S1_s_at	Xyloglucan endotransglucosylase/hydrolase protein 22-like	−2.76	0.0009
Cit.13424.1.S1_at	Putative cytochrome P450 protein homologue (*Arabidopsis thaliana*)	−2.68	0.0026
Cit.9523.1.S1_s_at	Translocator protein homolog	−2.67	0.0354
Cit.18045.1.S1_s_at	Ethylene-responsive transcription factor ERF023-like	−2.65	0.0011
Cit.30513.1.S1_x_at	Syringolide-induced protein 19-1-5 homologue (*Glycine max*)	−2.62	6.27E-06
Cit.9421.1.S1_s_at	Probable xyloglucan endotransglucosylase/hydrolase protein 23-like	−2.6	5.70E-05

### Analysis of Control vs. MFA

A comparison of gene expression in control citrus trees with MFA-treated citrus trees revealed 565 differentially expressed genes. 516 of these genes were upregulated, and 49 were downregulated. 2–56.5 fold changes were observed in the group of upregulated genes, and −2 to −3.8 fold change was noted in the downregulated group (Table 4 and the full comparative set is available in Supplementary File 1). This comparison revealed that genes related to defense regulation 1-aminocy clopropane-1-carboxylate synthase 1-like, Laccase-7-like, O-methyltransferase homologue, blue copper protein-like, BAHD acyltransferase At5g47980-like, cytochrome P450 83B1-like, phenylalanine ammonia-lyase-like, chitinase CHI1, ethylene-responsive transcription factor 1B-like, glucan endo-1,3-beta-glucosidase, basic isoform-like, and peroxidase C3-like were influenced. Downregulated genes included auxin-induced protein 22D-like, protein ECERIFERUM 1-like, regulator of chromosome condensation-like protein, purple acid phosphatase 8-like, expansin-A5-like, auxin-induced protein 22D-like, translocator protein homolog, and uncharacterized proteins and hypothetical proteins (Table 4).

**Table 4:** A sample of the differentially expressed genes in the control vs. MFA-treated groups.

Gene ID	Description	Fold change	*p*
*Upregulated*			
Cit.17838.1.S1_at	Uncharacterized LOC102619854	56.59	1.52E-05
Cit.3761.1.S1_x_at	Uncharacterized LOC102619665	39.39	5.42E-07
Cit.1441.1.S1_at	Acetyltransferase-like protein homologue (*Arabidopsis thaliana*)	33.12	3.13E-10
Cit.18037.1.S1_at	1-aminocyclopropane-1-carboxylate synthase 1-like	29.49	4E-11
Cit.2409.1.S1_s_at	laccase-7-like	22.38	1.4E-12
Cit.17291.1.S1_at	O-methyltransferase homologue (*Populus balsamifera subsp. trichocarpa x Populus deltoides*)	22.32	6.30E-09
Cit.17178.1.S1_x_at	Unknown	21.27	1.53E-07
Cit.3377.1.S1_at	Blue copper protein-like	20.96	6.6E-10
Cit.29411.1.S1_s_at	BAHD acyltransferase At5g47980-like	20.23	1.56E-10
Cit.7362.1.S1_at	Cytochrome P450 83B1-like	19.53	7.3E-10
*Downregulated*			
Cit.16432.1.S1_at	Hypothetical protein	−3.83	0.0002
Cit.9523.1.S1_s_at	Translocator protein homolog	−3.48	0.0339
Cit.3534.1.S1_s_at	Uncharacterized LOC102616771	−3.18	0.0153
Cit.21126.1.S1_s_at	Auxin-induced protein 22D-like	−3	5E-05
Cit.2093.1.S1_s_at	Expansin-A5-like	−2.66	0.0483
Cit.8231.1.S1_s_at	Purple acid phosphatase 8-like	−2.62	0.0022
Cit.29521.1.S1_x_at	Regulator of chromosome condensation-like protein homolog (*Arabidopsis thaliana*)	−2.6	0.0009
Cit.29521.1.S1_at	Regulator of chromosome condensation-like protein homolog (*Arabidopsis thaliana*)	−2.55	0.0014
Cit.38030.1.S1_at	Protein ECERIFERUM 1-like	−2.51	0.0006
Cit.17407.1.S1_at	Auxin-induced protein 22D-like	−2.46	6E-05

### Analysis of Infected vs. MFA+Infected

When gene expression in the infected trees was compared to the MFA+infected citrus trees, 909 differentially expressed genes were observed. 685 of these genes were upregulated, and 224 were downregulated in MFA+infected samples. 2–43.2 fold changes were observed in the group of upregulated genes, and 2 to −11.1 fold change was observed in the downregulated group. A sample of these genes is presented in Table 5, and the full comparative set is available in Supplementary File 1. The analysis revealed an upregulation in laccase-7-like, 1-aminocyclopropane-1-carboxylate synthase 1-like, BAHD acyltransferase At5g47980-like, blue copper protein-like, and genes associated with defense responses. This includes endochitinase 1-like, glucan endo-1,3-beta-glucosidase, basic isoform-like, ethylene-induced esterase, phenylalanine ammonia-lyase-like, and peroxidase 15-like proteins. There were also a number of unknown and uncharacterized genes upregulated. Among the most downregulated genes were chalcone synthase-related genes and homologues, a Protein ECERIFERUM 1-like, glycine-rich cell wall structural protein 1-like and GDSL esterase/lipase EXL1-like, and putative copia-like retrotransposon protein homologue (Table 5).

**Table 5:** A sample of the differentially expressed genes in the infected vs. MFA+infected groups.

Gene ID	Description	Fold change	*p*
*Upregulated*			
Cit.2409.1.S1_s_at	Laccase-7-like	43.29	4.46E-14
Cit.1441.1.S1_at	Unknown	43.23	1.43E-10
Cit.26572.1.S1_at	Uncharacterized LOC102607820	37.15	2.50E-08
Cit.3761.1.S1_x_at	Uncharacterized LOC102619665	32.54	3.29E-07
Cit.18037.1.S1_at	1-aminocyclopropane-1-carboxylate synthase 1-like	31.54	2.58E-11
Cit.29411.1.S1_s_at	BAHD acyltransferase At5g47980-like	27.46	4.49E-11
Cit.26572.1.S1_s_at	Uncharacterized LOC102607820	24.17	9.71E-09
Cit.17178.1.S1_x_at	Unknown	22.58	1.72E-07
Cit.3377.1.S1_at	Blue copper protein-like	21.58	3.80E-10
Cit.17838.1.S1_at	Uncharacterized LOC102619854	21.44	9.41E-05
*Downregulated*			
Cit.35493.1.S1_s_at	Uncharacterized LOC102612783	−11.1	0.0364
Cit.21179.1.S1_at	Chalcone synthase 2 homologue (*Citrus sinensis*)	−7.53	0.0003
Cit.8600.1.S1_x_at	Chalcone synthase 2 homologue (*Citrus sinensis*)	−5.22	0.0007
Cit.19520.1.S1_s_at	Chalcone synthase 2-like	−4.7	0.001
Cit.38030.1.S1_at	Protein ECERIFERUM 1-like	−4.5	3.57E-05
Cit.30458.1.S1_s_at	Chalcone synthase 2-like	−3.97	0.0016
Cit.19520.1.S1_x_at	Chalcone synthase 2-like	−3.89	0.0014
Cit.34812.1.S1_s_at	Glycine-rich cell wall structural protein 1-like	−3.8	0.0052
Cit.39287.1.S1_s_at	GDSL esterase/lipase EXL1-like	−3.7	0.0037
Cit.27421.1.S1_at	Putative copia-like retrotransposon protein homologue (*Oryza sativa*)	−3.52	0.0009

### Common Upregulated Genes Shared by MFA-Treated Trees

There were 403 genes directly influenced by MFA, following the Venn analysis of intersecting genes between control vs. MFA and infected vs. MFA+infected (Figure 3). The genes uniquely responsive to the MFA treatment are highlighted here. Among the top upregulated genes in the two sample sets include Laccase-7-like which plays a role in lignin polymerization and anthocyanin degradation. The gene BAHD acyltransferase At5g47980-like was also upregulated in the two groups. BAHD acyltransferases are involved in the production of defense and anti-herbivory compounds. The blue copper protein-like gene, which is involved in redox reactions, was also highly upregulated in the two groups possibly reflective of the higher Cu concentrations in the MFA-treated trees.

Increased expression was recorded in several plant hormone response genes which may indicate the type of defense response induced by MFA. This included genes associated with ethylene concentration including ACC oxidase, 1-aminocyclopropane-1-carboxylate synthase 1-like, ethylene-responsive transcription factor 1A-like, and protein reversion-to-ethylene sensitivity1-like. Abscisic acid-associated genes were among the groups of upregulated genes and included CYSTM1 family protein A-like, exocyst complex component EXO70B1-like, zeaxanthin epoxidase, chloroplastic-like, and threonine dehydratase biosynthetic chloroplastic-like protein. There were also several indole-3-acetic acid (IAA)-related genes downregulated in the leaf tissue in response to MFA which may have indicated a reduction in IAA production in the trees. These included a IAA-amino acid hydrolase ILR1-like 6-like, probable indole-3-acetic acid-amido synthetase GH3.1-like, and a auxin-induced protein 22D-like.

Among the genes that were influenced, increased expression was observed in multiple PR genes such as PR2, PR4, and PR5; genes involved in the phenylpropanoid biosynthesis. These included shikimate O-hydroxycinnamoyltransferase-like, 4-coumarate–CoA ligase-like 10-like, and feruloyl CoA ortho-hydroxylase 1-like genes. Upregulated lignin-specific genes included caffeoyl-CoA O-methyltransferase-like, cinnamoyl-CoA reductase 1-like, and caffeic acid 3-O-methyltransferase-like genes.

A number of genes associated with oxidative responses were also increased in response to MFA. This includes ROS regulators such as peroxidases. Genes associated with respiratory burst oxidase homolog protein D-like, cinnamate 4-hydroxylase CYP73, and peroxisomal membrane protein PMP22-like were also upregulated. Several transcriptional activators increased in response to MFA. These included WRKY transcriptional factors, myb-related proteins, NAC domain-containing proteins, scarecrow-like protein 14-like, and transcription factor RAX2-like. Genes associated with cell growth, cell remodeling, and cell structure were also upregulated. These included extensin, LOB domain-containing protein 11-like, receptor-like serine/threonine-protein kinase SD1-8-like, QWRF motif-containing protein 8-like and metalloendoproteinase 1-like, probable galacturonosyltransferase-like 10-like, early nodulin-93-like, and alkaline/neutral invertase CINV2-like.

Genes associated with alkaloid biosynthesis were also upregulated in response to MFA. These included cannabidiolic acid synthase-like 2-like, anthranilate synthase component I-1, chloroplastic-like, tropinone reductase-like 1-like anthranilate N-methyltransferase-like, taxadien-5-alpha-ol O-acetyltransferase-like, and codeine O-demethylase-like. Transport genes were among a series of upregulated genes in the citrus trees in response to MFA. These are involved with ABC transporters and iron transport. Other genes (Supplementary File 1) are possibly involved in brassinosteroid biosynthesis, anthocyanin synthesis, lipase activity, senescence, elicitor and wound responses, ubiquidation, SAR, homeostasis, amino acid synthesis, endopeptidase inhibition, and energy metabolism.

## Discussion

This study examined the impact of a microbial preparation, MFA, against HLB in young citrus trees. The study assessed four experimental groups: control, infected, MFA treated, and MFA treated + infected. The results presented here indicate that MFA increased the transcriptional activity of citrus defense mechanisms, elevated Cu concentrations in citrus leaf tissue, and stabilized HLB infection in treated plants (Figure 1, Table 1).

Following monthly treatments of MFA over the experimental period (Figure 1), there was a consistent increase in disease severity over time in infected trees which is consistent with previous observations (Li et al., 2018). A DI reduction of 13.3% in MFA-treated trees was observed at 20months, but this infection rate did not significantly deviate from the untreated infected group (Figure 1B). Monitoring of the distribution of change in DI revealed that MFA, when all trees and infected trees were considered, resulted in significant stabilization of disease progression in the MFA-treated groups (Table 1). PR2 was significantly increased 2h after MFA treatment (Figure 2A), and PAL was also significantly increased 6h after treatment in response to MFA (Figure 2B). In addition to this, a transcriptomic assessment revealed an array of defense mechanisms that were upregulated in response to MFA. The previous work examined the impact of plant defense elicitors on HLB in a field evaluation. Li et al. (2015a) and Li et al. (2018) found a consistent reduction in disease severity when β-aminobutyric acid (BABA), benzothiadiazole (BTH), ascorbic acid (AA), and salicylic acid (SA) were applied to citrus. Induction of PR2 was observed after BABA and BTH applications, suggesting its implication in the defense against HLB.

The significant increases in Cu concentrations of citrus leaves following treatment with MFA (Table 2) observed in this study can be attributed to the Cu in the application which significantly increased the mineral in healthy and infected trees to 32.4 and 26.8ppm, respectively. Copper nutrition is critical in citrus development. Its deficiency is commonly associated with rapid growth in non-bearing trees following periods of high nitrogen fertilizer usage, and this leads to distorted leaf and twig growth and visual leaf chlorosis (Yruela, 2009; Hippler et al., 2017). Cu is poorly mobile in the phloem. Its application is recommended during periods of new vegetative growth (Marschner, 2012; Hippler et al., 2017) and should be regularly applied as foliar and ground fertilizer (Hippler et al., 2018). Cu is an important cofactor for many enzymes such as superoxide dismutase, amino oxidase, laccase, blue copper protein, and plastocyanin, but it also plays an important role for the transcription of protein trafficking machinery and oxidative phosphorylation (Yruela, 2005, 2009). Applications that increase Cu uptake in citrus could therefore be deemed valuable for agronomic purposes. Cu as a phytosanitary application has been used in the control of pathogens for many years (Russell, 2005; Hippler et al., 2017) and is considered a potent antimicrobial element (Vincent et al., 2018). The Cu portion of MFA may have contributed to the stabilization of HLB DI. In an investigation into the impact of micronutrients on HLB in *C. sinensis*, Da Silva et al. (2020) examined the use of copper hydroxide fertilizer on HLB in citrus. These authors reported that although leaf Cu concentrations reached 20ppm, there was no significant impact on HLB concentrations. The authors also reported that individual Cu fertilizer treatments helped mitigate the impact of HLB on starch metabolism a fundamental issue in trees infected with the disease. For this reason, MFA could potentially play a role in alleviating HLB symptoms by delivering higher Cu concentrations in infected citrus trees. However, the added elicitation of defense responses by MFA may have contributed to stabilization of disease progression. A previous investigation which compared CuSO_4_, MFA without CuSO_4_ and MFA reported that MFA had a significant impact on the induction of defense-related genes and on the control of powdery mildew in wheat (Twamley et al., 2019). The evidence presented suggests that MFA as a combined formulation (fermentation media and CuSO_4_) gave the greatest control of disease and was the most effective at priming defense-related responses. This observation was further supported when MFA demonstrated greater antifungal activity against *Zymoseptoria tritici* compared to individual treatments of CuSO_4_ and MFA without CuSO_4_ at reduced concentrations *in vitro* (Twamley et al., 2021). Twamley et al. (2021) also indicated that MFA-treated plants resulted in grain yield and quality improvements in healthy MFA-treated plants. The authors suggest that this could be a function of peptide or amino acid complexes that might have greater bioactive properties and that could help limit the oxidative damage caused by Cu in plants. This suggests that the microbial preparation with CuSO_4_ (MFA) possibly responds better than CuSO_4_ or the fermentation media in isolation. The formulation may have a unique synergistic mechanism as a combined product. This may suggest that in the current study, the total preparation is conferring the benefits observed in the data for citrus and not just the CuSO_4_ or fermentation media in isolation. However, a more comprehensive study should be conducted to confirm this. Excessive Cu use will continue to be regulated against due to its negative environmental impact. Issues such as accumulation and the selection of Cu resistant pathogens in the natural environment are of primary concern (Lamichhane et al., 2018). Environmentally safe microbial preparations that can prime plant natural defenses may play a role in reducing the use of Cu as a phytosanitary application.

Transcriptomic analysis gives an insight into what cellular processes are dormant or active in response to either a disease, challenge, or a nutritional treatment. In the control vs. infected trees, there were several differentially expressed genes associated with HLB infection. In previous studies conducted in greenhouses, HLB infection significantly impacted between 604and 633 genes (245–589 upregulated and 22–350 downregulated; Kim et al., 2009; Mafra et al., 2013; Fu et al., 2016; Hu et al., 2017). In the present study, HLB infection impacted a total of 171 genes (150 upregulated and 21 downregulated), in a field infection model. The most common issues associated with HLB are alterations in transcriptional processes related to defense, oxidative stress, carbohydrate metabolism, cell structure and organization, transcription factors, hormone signaling, and phloem blockage (Kim et al., 2009; Albrecht and Bowman, 2012; Mafra et al., 2013; Martinelli et al., 2013; Fu et al., 2016; Hu et al., 2017). Each of these processes are believed to contribute to the rapid development of disease symptoms, ultimately leading to declining tree health and death. Similar patterns for many of the previously reported correlations (Table 3 and Supplementary File 1) were observed in this study. Peroxidase, thaumatin-like protein, beta-galactosidase 1-like, hypersensitive-induced response protein 2-like, and elicitor-induced gene product homologue were upregulated in response to HLB infection. These are indicators of defense and oxidative stress-related responses in citrus and have previously been associated with HLB infection (Kim et al., 2009; Fu et al., 2016). Pathways associated with secondary metabolite biosynthesis were also upregulated, including multiple PAL genes, flavanone synthase genes, isoflavone 4′-O-methyltransferase-like gene, (E)-beta-farnesene synthase, and a trans-resveratrol di-O-methyltransferase-like gene. Phenylpropanoids are both associated with defense and the syntheses of flavonoids and terpenes which both play a role in the attraction of pollinating insects, defense against herbivories, and in the establishment of disease resistance. Previous studies have indicated that HLB impacts phenylpropanoid and terpene synthesis (Albrecht and Bowman, 2012; Fu et al., 2016; Hu et al., 2017).

Genes involved with carbohydrate metabolism and cell wall modification were also significantly impacted. Early nodulin, extensins, probable polygalacturonase, sucrose synthase, xyloglucan endotransglucosylase/hydrolase, cellulose synthase, and pectinesterase inhibitor were included in the group of upregulated genes responding to HLB infection in this study. The previous work has identified transcriptomic signatures that demonstrate the impact of HLB on carbohydrate metabolism (Fu et al., 2016, Hu et al., 2017) where expansins, xyloglucan endotransglucosylase/hydrolase proteins, cellulose synthesis, and pectinase-related genes were previously reported as differentially impacted in response to HLB. Genes involved in hormone signaling were also significantly impacted between control and HLB-infected trees; specifically, three gibberellin-regulated genes and a HVA22-like gene were upregulated in the HLB-infected tissue. Auxin-responsive protein, brassinosteroid-regulated gene, ethylene-induced esterase homologue, and an ethylene-responsive transcription factor were all downregulated. Phytohormones are believed to be impacted due to the cellular processes that are distorted as a result of changes in cell growth and defense responses. Zheng and Zhao (2013) saw similar changes in phytohormone production including auxin-, ethylene-, and gibberellin-related genes which may play a role in the mediation of citrus responses to HLB.

Many of the genes impacted following MFA treatment are associated with a strong localized and systemic defense responses. These included direct defense genes and numerous genes involved in the regulation of biotic defense responses. The defense stimulatory response may have supported the 13.3% reduction in DI (Figure 1). Disease progression was also significantly stabilized (Table 1) in MFA-treated trees. The transcriptome analysis revealed an increase in many direct defense-related enzymes but also a series of pathways involved in secondary metabolite biosynthesis and the production of antimicrobial compounds (Tables 4 and 5, and Supplementary File 1). Microbial elicitors have been successful in the past in the induction of resistance to bacterial, fungal, and oomycete pathogens (Thakur and Sohal, 2013). MFA contains both bacterial and yeast fermentation media, and both components may play a role in initiating gene transcription responses and have benefited plants in the past (Wiesel et al., 2014). The application of a yeast suspension to Arabidopsis saw the development of resistance to both *Pseudomonas syringae* and *Botrytis cinerea* infections (Raacke et al., 2006). The study also uncovered the ability of the yeast application to stimulate the expression of several plant defense systems including SAR, detoxification, and the jasmonate/ethylene pathways (Raacke et al., 2006). Yeast cell wall extracts are also well-documented inducers of plant defense, observed to regulate plant stomatal closure and mediate ROS responses following their use in various plant models (Khokon et al., 2010; Wiesel et al., 2014). A study that investigated ROS generation in rice in response to a N-acetylchitooligosaccharide demonstrated its role in plant defense elicitation (Kuchitsu et al., 1995). In Arabidopsis, chitin elicitors also increased ROS generation while contributing to both fungal and bacterial suppression (Egusa et al., 2015). The benefits of bacterial derived elicitors for plants have also been reported. They trigger induced systemic resistance (ISR) in plants and have other secondary benefits to plant health (Choi and Klessig, 2016; Ek-Ramos et al., 2019). ISR is understood to be important in cell wall thickening or destruction of infected cells, which helps cut off nutrients and access to invading pathogens. This has been effective against a range of plant pathogens in greenhouse and field settings (Lugtenberg et al., 2002; Aloo et al., 2019).

Phenylpropanoids produce an array of secondary metabolites derived from intermediates of the shikimate pathway (Fraser and Chapple, 2011). They contribute to a plant’s response to biotic and abiotic stimuli. PAL catalyzes the non-oxidative deamination of phenylalanine to trans-cinnamate and directs carbon flow from the shikimate pathway to general phenylpropanoid biosynthesis (Vogt, 2010). Phenylpropanoids are also understood to be involved in a plants overall defense strategy. They form preformed defenses, inducible defense responses, physical barriers and act as signaling molecules (Dixon et al., 2002). They are also well documented in plants in response to pathogens and in the development of resistance to disease. This data provides evidence that MFA may result in the beneficial upregulation of the phenylpropanoid pathways, and several phenylalanine ammonia-lyase-like genes were upregulated and complimented with shikimate O-hydroxycinnamoyltransferase-like, 4-coumarate--CoA ligase-like, and feruloyl CoA ortho-hydroxylase. There were significant increases in the lignin synthetic genes including caffeoyl-CoA O-methyltransferase-like, cinnamoyl-CoA reductase 1-like, and caffeic acid 3-O-methyltransferase-like which could be indicative of enhanced lignin formation. Laccase-7-like and blue copper protein was among the top 10 highest upregulated genes in healthy and infected trees where MFA was applied (Tables 4 and 5). Laccases are multi-copper enzymes that catalyze substrate oxidation and that reduce molecular oxygen to water. Laccases are also involved in Cu-facilitated lignification of cell walls contributing to defense, structure, and rigidity (Janusz et al., 2020). Xu et al. (2019) indicated that citrus laccases respond to environmental stress and are involved in lignin synthesis, and Cu ions are bound in several sites in laccases by Type 1 blue copper proteins (Printz et al., 2016), making blue copper proteins essential in the formation and function of laccases. Based on the transcriptomic evidence provided in this study, MFA potentially may play a crucial role in the activation of the phenylpropanoid pathway and facilitates lignin formation in citrus cell walls.

In this study, several PR genes were upregulated by MFA, including chitinase, endochitinase, endo-1,3-beta-glucosidase, osmotin, peroxidases, and thaumatin-like proteins (PR2, PR3, PR5, PR8, and PR9). Plants produce several direct defense mechanisms when initially challenged by pathogens. The importance of PR gene expression in citrus biotic responses has previously been reported (Campos et al., 2007) and highlights their role in pathogen defense responses. PR gene expression was expected to be upregulated as the data generated in the initial RT-qPCR experiment revealed significant upregulation of PR2 prior to microarray transcriptomic analysis (Figure 2).

Plant hormones are known to be important in plant growth, development, and cell signaling. They are also understood to be crucial in plant defense responses (Bari and Jones, 2009). In this study, there were several differentially expressed genes involved with plant hormone synthesis and regulation in response to MFA. This included genes associated with IAA, ethylene, and abscisic acid. There were also a small number of genes associated with brassinosteroids, salicylic acid, and jasmonic acid. Ethylene is produced from methionine by ACC synthase (ACS) and ACC oxidase (ACO) from its precursor 1-aminocyclopropane-1-carboxylic acid (ACC). The results presented herein revealed that S-adenosylmethionine synthase-like genes ACC synthase and a homologue were upregulated in response to MFA. Two ACC oxidases and ACC oxidase homologues were upregulated in response to MFA. Ethylene-responsive transcription factor-like genes 1A (2), 1B (2), 5, and 113 were also upregulated in MFA-treated plants. These ethylene-responsive transcription factors bind to the GCC-box pathogenesis-related promoter responding to pathogens and increasing the expression of PR genes (Zhou et al., 1997; Fujimoto et al., 2000; Sakuma et al., 2002).

## Conclusion

This work provides evidence that MFA when applied to infected and healthy citrus trees increases Cu concentration, stabilizes HLB disease progression, and increases the expression of defense-related genes 2h after its application in a field setting. Transcriptomic assessment revealed that MFA increased the expression of 565 genes in healthy citrus trees and 909 genes in HLB-infected trees. This included traits associated with plant growth, development, and defense. There are very few successful applications that can be integrated into citrus production systems to help manage HLB. Provided herein are promising data suggesting a microbial preparation may help slow the progression of HLB in a field scenario. Further work is required to determine the mode of action of the application. Due to the limits in phytosanitary use of Cu, future work will address whether the efficacy of CuSO_4_ contained in MFA is enhanced by its microbe-derived fraction, thus allowing for a reduced Cu input in the environment. Work will also aim to determine the impact of MFA on mature HLB-infected citrus trees in multiyear studies. MFA may also play a role in the management of other problematic pathogens; future research will aim to determine whether MFA has a broader application in agronomy.

## Data Availability Statement

The original contributions presented in the study are publicly available. This data can be found here: ArrayExpress accession E-MTAB-10919.

## Author Contributions

RL, KDo, SB, and KDa designed set up and managed the experiment and its logistics. RL, KDo, UC, WS, and ED applied treatments, sampled the trial, and prepared the samples for mineral and qPCR analysis. RL, KDo, and KE prepared and ran the transcriptomic samples. RL, RM, SB, KH, and KDa managed and supported the overall study. RL, KH, and KDa prepared and wrote the manuscript. All authors contributed to the interpretation of the data prior to preparing the manuscript. All authors contributed to the article and approved the submitted version.

## Funding

This work was funded in full by Alltech and Alltech Crop Science. Alltech provided the research facilities and financial support for this work.

## Conflict of Interest

All the authors were paid employees of Alltech during their contributions to this project. Alltech innovate and manufacture crop input products for the agronomic industry.

## Publisher’s Note

All claims expressed in this article are solely those of the authors and do not necessarily represent those of their affiliated organizations, or those of the publisher, the editors and the reviewers. Any product that may be evaluated in this article, or claim that may be made by its manufacturer, is not guaranteed or endorsed by the publisher.
